# Coping or adapting? Experiences of food and nutrition insecurity in specialised fishing households in Komodo District, eastern Indonesia

**DOI:** 10.1186/s12889-021-10248-3

**Published:** 2021-02-15

**Authors:** Emily Gibson, Natasha Stacey, Terry C. H. Sunderland, Dedi S. Adhuri

**Affiliations:** 1 Research Institute for the Environment and Livelihoods, Charles Darwin University, Ellengowan Drive, Darwin, Northern Territory Australia; 2 Department of Forest and Conservation Sciences, University of British Colombia, 2424 Main Mall, Vancouver, Canada; 3 Centre for International Forestry Research, Bogor, Indonesia; 4 Research Centre for Society and Culture, Indonesia Institute of Sciences, Jl.Jend Gatot Subroto 10, Jakarta, Indonesia

**Keywords:** Specialised fishers, Small-scale fisheries, Food and nutrition security, Dietary diversity, Indonesia

## Abstract

**Background:**

There is growing recognition of the need for fish to be better integrated into nutrition-sensitive strategies for addressing malnutrition. Fish are overwhelmingly produced by the small-scale sector, which supports food and nutrition security directly through the provision of fish and indirectly through the generation of income which can be used to purchase other desired foods. However, there has been relatively little research on the extent of food and nutrition security in specialised fishing communities. This study assessed food and nutrition security among households in specialised fishing communities in Komodo District, eastern Indonesia.

**Methods:**

We assessed the seasonal nutrition quality of household diets using the Food Consumption Score for nutritional analysis and food insecurity using the Household Food Insecurity Access Scale in 66 households across three communities, using a modified cluster sampling strategy. We calculated and generated descriptive statistics for these indicators with Microsoft Excel and ran a logistic generalized linear mixed model to determine factors associated with severe food insecurity using SPSS. We used semi-structured interviews and focus group discussions to understand perceptions of, change over time, and strategies for dealing with food shortfalls.

**Results:**

While most households have acceptable access to nutritious foods, especially protein and heme iron-rich foods, nearly one half of households consumed vitamin A rich foods on less than 3 days of the 7-day recall period in either season. More than half of households reported experiencing a moderate or severe level of food insecurity, with higher food insecurity in the wet season. Low maternal education (OR: 3.8, 95%CI 1.5–9.9) and lower household wealth (OR: 0.5, 95%CI 0.3–0.9) were found to be associated with a severe level of food insecurity. Household’s consumptive and non-consumptive response strategies reflect adaptation to chronic food insecurity but are nutritionally and economically unsustainable.

**Conclusion:**

Households in specialised fishing communities in Komodo District consumed diets with low diversity and experienced high levels of food insecurity. There is a need for culturally-appropriate nutrition-sensitive strategies to enhance food and nutrition security in vulnerable fishing communities.

**Supplementary Information:**

The online version contains supplementary material available at 10.1186/s12889-021-10248-3.

## Background

Food insecurity is a pressing global issue and contributes to malnutrition in all its forms (undernutrition, micronutrient deficiency, and overweight and obesity) through the complex interplay of food and non-food factors that affect a person’s nutritional status [39]. In 2018 an estimated 820 million people were classified as undernourished and two billion people were affected by micronutrient deficiency or ‘hidden hunger’ [40]. “Hidden hunger occurs when the quality of food people eat does not meet their nutrient requirements, so the food is deficient in micronutrients such as the vitamins and minerals that they need for their growth and development” [100]. Hidden hunger can occur in those consuming too little food or in those consuming too much energy-dense nutrient poor food. Globally, undernutrition was the underlying factor in 45% of all deaths of children under 5 years of age in 2011 [7], and suboptimal diets are responsible for more adult deaths than any other risks [44]. The most common nutritional deficiencies are of iron, zinc and vitamin A, and multiple micronutrient deficiencies can occur together in the same population [78]. The WHO [101] recommends consumption of a healthy diet to avoid nutritional deficiencies and non-communicable diseases. A healthy diet comprises an appropriate energy intake, increased intake of fruits, vegetables, legumes, nuts and wholegrains, moderate intake of animal-source foods such as fish, and reduced intake of ultra-processed foods, to meet the nutrient requirements of all age groups, including those with special nutrition needs, and is adapted to the local context and culture [77, 101, 104]. Ultra-processed foods “are typically high-energy-dense products, high in sugar, unhealthy fats and salt, and low in dietary fiber, protein, vitamins and minerals” ([76], p.1). They are generally manufactured in an industrial setting, and in developing countries are widely available as single-serve packaged sweet or savory snack foods. In the context of the multiple burden of malnutrition, and the complex challenges of a growing global population and climate change, there is growing recognition that healthy diets must also be produced by sustainable food systems so as to ensure food and nutrition security for present and future generations [36, 52, 54, 104].

Fish, and other edible marine resources (e.g. bêche-de-mer, octopus, sea shells, shrimp; hereafter “fish”), have largely been overlooked in discussions of food-based strategies to address malnutrition in the context of sustainable food systems [10, 53, 62, 63, 68, 91]. Fish are a nutrient-dense animal-source food which provides 3.2 billion people with almost 20% of their average per capita intake of animal protein, with consumption higher in Indigenous fishing communities and small-island developing States [17, 22, 38]. However, the nutritional value of fish extends beyond protein, encompassing long-chain polyunsaturated fatty acids, micronutrients such as vitamins A, B12 and D, and minerals including calcium, phosphorous, iodine, zinc, bioavailable heme iron, and selenium [5, 11, 85]. Fish are an invaluable addition to plant-based diets because they enhance the bioavailability of non-heme iron and zinc from other foods consumed in the same meal [69, 70]. In developing countries, the small-scale fisheries sector, operating across marine, inland and aquaculture systems, employs an estimated 33.1 million fishers on a full- or part-time basis, with an additional 2–3 people for every fisher employed in postharvest activities; half of these workers are women [72]. The sector provides a safety net function, with countless others engaged in fishing activity on an occasional and seasonal basis [6]. These activities contribute directly to food and nutrition security through the provision of fish for home consumption and indirectly through the generation of income which can be used to purchase other foods, including staples and lower-value fish, and to pay for other goods and services.

‘Fish as food’ is an important lens for thinking about the sustainability of fisheries and fishing communities [5, 63]. Studies at a global scale indicate that in some countries with a high malnutrition burden redirecting a small portion of marine finfish landings to local rather than export markets could have a meaningful impact on the nutritional value of local diets, especially for children under the age of five [51]. Studies at a local scale tend to focus on the apparent availability of fish, particularly in the context of marine governance and sustainability innovations, rather than on how fishing contributes to food and nutrition security within local fishing communities. Kawarazuka and Bene [58] mapped evidence of these contributions across three pathways (income, consumption and distribution), but noted that more research was required to quantify the linkages between fish-related livelihoods and nutrition. Moreover, fishing households face heightened risk of food insecurity as they are at risk of direct and trading entitlement failures because their food and nutrition security depends on the consumption of the food they produce (i.e. fish) *and* its sale to obtain other foods [14]. These risks may be higher in specialised fishing households and communities that are constrained by limitations of access to productive resources, infrastructure, economic and institutional capacity, and where the socio-cultural ties to fishing as a way of life are strong [20, 34, 60].

In this paper, we present and analyze empirical data from a mixed methods exploratory case study of food and nutrition security in three specialised fishing communities in eastern Indonesia. We use food consumption frequency data to assess nutritional quality of diets at the household level, assess the prevalence and severity of food insecurity (access), and identify strategies used by households to manage during times of food insufficiency. The study contributes to the literature by identifying the vulnerability of specialised fishing households to chronic food insecurity and the need for cross-sectoral nutrition-sensitive programmes to improve food security in similar communities in the tropical coastal seascape.

## Methods

### Study area

Indonesia has the largest reef-associated population in the world, with nearly one third of its’ 270 million-strong population living within 10 km of the coast [15, 105]. The country is the world’s second largest producer of marine fish [38], with most of this catch harvested by small-scale fishers. Government data indicates that the majority of targeted commercial fish stocks are fully exploited or overexploited [74], and fisheries production is threatened by destructive and unsustainable fishing practices, land-based pollution, as well as climate variability [4, 15, 18]. Indonesia has demonstrated a high-level political commitment to improving undernutrition, yet it is ranked 73rd out of 119 countries in the Global Hunger Index [95], the national prevalence of stunting in children under 5 years of age is 36.4, and 28.8% of women of reproductive age are anaemic [102].

This research was undertaken in Komodo District, West Manggarai Regency, in the Province of Nusa Tenggara Timur (NTT). Fisheries are an important contributor to the local economy. Fishing and trading activity are dominated by Indigenous Sama-Bajau and Bugis households who live in settlements near the main fish landing site at Labuan Bajo and on numerous nearby islands. Fishing communities have slightly different fishing profiles, targeting different species using different gear, with a network of patrons and middlemen providing connections to local, domestic and even high-value export markets. While it is typically men who go to sea, women are involved in activities across the local value chain. However, women’s involvement is predominantly in unpaid pre- and post-harvest activities that support their fisher spouse and extended family, or in seasonal post-harvest work for which they are remunerated with fish. The gendered division of labor reflects both Indigenous culture and Islamic religious observance.

In 2017, 20% of the District’s population were classed as poor [13]. A food security and vulnerability assessment conducted in 2015 by the Provincial government, in conjunction with the World Food Programme, considered Komodo District ‘food secure’ [46]; however only one third of households had access to electricity, 46% had access to clean water and 49.31% of children under five had stunted growth [46]. A recent local study relying on food balance sheet data found that diets were lacking diversity [82].

Our case study sought to provide a holistic assessment of food and nutrition security in specialised fishing communities. Our research was guided by a comprehensive review of the literature and Kawarazuka and Bene's [58] pathways framework, which is in itself based on the UNICEF [93] ‘determinants of nutritional status’ (see Table S1). The three pathways (income, consumption and distribution) in which fish may contribute to food and nutrition security are mapped across the basic, underlying and immediate causes of nutritional status (or outcomes). We purposively selected three villages for our case study, each situated on a different island located between seven and 14 km from Labuan Bajo. There was a high prevalence of fishing livelihoods in each of the villages, but the villages differed based on their structure (formal / informal village status), geography (small rock outcrop to large savannah island, with/without fringing reef), population size, and location. The first component of our research (undertaken simultaneously) explored the contribution of fish to the diets of women of reproductive age and infants and young children [45]. We found that while fish was a central component of women’s diets, over 50% of mother-child pairs failed to meet the minimum recommended dietary diversity. Furthermore, the introduction of fish to young children’s complementary diets was delayed due to fears and other concerns. This paper assesses the extent of food and nutrition security at the household level and responses to food insecurity.

### Data collection

The research applied mixed methods, with a combination of qualitative and quantitative research activities suited to an interdisciplinary exploration of food and nutrition security [30]. All field work was conducted between September 2017 and May 2018; research activities were conducted during five trips to each village, with activities sequenced to allow for comprehensive coverage of key thematic areas identified in the research framework as well as to allow for in-depth exploration of emerging themes (see Table 1) [25]. The extended field work period, and repetition of certain modules of the household questionnaire, also allowed for the development of familiarity and rapport between the research field team and community members [59]. This team comprised the first author and three locally-engaged research assistants who acted as facilitators for research activities and translators. Research assistants received training on key elements of the research framework and the data collection tools used. The household questionnaire and interview / discussion guides were prepared in advance and reviewed as part of the training to ensure key concepts were accurately translated and that appropriate local terms were used. All activities were implemented in Indonesian or the local dialect (Bahasa Bajau), in which case one research assistant acted as a secondary translator.

**Table 1:** Summary of field site characteristics and research activities

Research activities	Field sites				Total
	FS 1	FS 2	FS 3	Labuan Bajo	
Population / households ^a^	1860 / 477	604 / 152	242 / 56		2706 / 685
Eligible households ^b^	186	52	25		263
Household survey					
January 2018	36	20	10		66
April 2018	34	15	10		59 ^c^
Interviews					
- Key informant	5 (3 M; 2 W)	3 (2 M; 1 W)	2 (1 M; 1 W)	6 (2 M; 4 W)	16 (8 M; 8 W)
- Semi-structured	12 (0 M; 12 W)	6 (0 M; 6 W)	4 (0 M; 4 W)	2 (0 M; 2 W)	24 (0 M; 24 W)
Focus group discussions	5 (8 M; 29 W)	2 (4 M; 10 W)	2 (6 M; 5 W)		9 (18 M; 44 W)

*M* man, *W woman*

^a^ estimate from village census information, October 2017; ^b^ estimate based on information obtained from local health volunteers; ^c^ participation in the second round of the household survey was reduced, with two households declining to participate and the remaining households absent from the village during the survey period

The introduction to research activities included an explanation of the purpose of the study, and later a summary of activities undertaken to that point in time, and how the instant activity contributed to the inquiry. Semi-structed interviews and focus group discussions were conducted to gather information about historic and contemporary livelihood activities across seasons, women’s household and community roles, historic and contemporary meal patterns, and experiences of food insecurity and coping/adapting strategies for times of food insufficiency. Semi-structured interviews were conducted with key informants from the Regency’s food security and fisheries agency and health department, community health staff and village leaders, and with community members (women, men, and those involved in fisheries). Key informants were identified based on their role (e.g. community nurse/midwife) and they subsequently identified potential community participants based on topics of interest. Additional participants were identified through purposive (i.e. to ensure coverage of different fishing activities) and snowball sampling [29]. Interviews were conducted in the participant's home or office and lasted for 30–75 min. Focus group discussions were held with community members identified by key informants. An initial focus group discussion in each community exploring seasonal food insecurity comprised a mix of women and men, whereas subsequent groups were targeted depending on the topic and themes to be explored and were conducted separately with either women or men (e.g. ‘mothers with young children’, ‘males (open age) who fish’). Focus group discussions were held in village meeting places and lasted for approximately 45 min. Interviews were recorded unless the participant declined (*n* = 2). The audio recording was subsequently transcribed and translated by a research assistant. In the case of interviews that were not recorded and focus group discussions, detailed notes were taken at the time of the activity and then reviewed collaboratively by the field research team to ensure that key themes, discussion points and contributed stories were accurately documented. This, together with debriefing of research activities with the research team, supported bracketing throughout the study [92]. Key informants with public health roles provided feedback on preliminary findings from the household questionnaire (see below). We applied the principle of saturation in assessing topic coverage, while multiple sequential research activities allowed for triangulation [29, 87].

A household survey using a structured questionnaire was conducted in each of the island villages. Eligible households were those with at least one women of reproductive age (aged 18 to 49 years, hereafter referred to as women or mothers) and a child aged between 6 months and 5 years of age. The questionnaire comprised three parts: (a) respondent and household socio-economic data, including men’s and women’s income-generating livelihood activities, asset ownership, and estimated monthly income and food expenditure; (b) food and nutrition security, including the Food Consumption Score Nutritional Quality Analysis [96, 97] and Household Food Insecurity Access Scale (HFIAS) [24] (see Data analysis below), both of which have been validated as indicators of household food insecurity in different settings, including Indonesia [3, 23, 103]; and (c) household decision-making and health and nutrition knowledge. The questionnaire comprised a combination of standard modules (e.g. access to drinking water and sanitation, food and nutrition security indicators) which were locally-adapted through discussion with key informants in accordance with relevant guidance documents, together with questions relevant to the study. The questionnaire is available as Supplementary Material to Gibson et al. [45]. The full questionnaire was conducted in January 2018 during the wet season and part (b) was repeated with the same households in late April during the dry season.^1^ This repetition allowed for the capture of seasonal variations in access to, availability of and consumption of fish and other foods. In total, 66 households participated in the survey. The sample size was determined based on the estimated prevalence of child stunting for Komodo District (49.31% [46]) and the estimated number of eligible households, sufficient to give a standard error of 12.5% at a 95% confidence interval [26]. A list of eligible households was prepared with the assistance of local health staff and, as Indonesian villages are administratively divided into sections (RTs), five randomly selected eligible households from each section were surveyed. The survey took between 45 min and 1.5 h to complete. The survey was piloted in a similar mainland community and minor amendments were made to simplify rating scale questions.

### Data analysis

#### Food consumption score nutritional quality analysis

Data on household food consumption was collected based on the module developed by [96, 97]). The module was pre-adapted using information about contemporary dietary patterns collected in key informant interviews, focus group discussions and a market survey of locally available foods. Additional questions were included about the type of fish consumed. A list method, in which the respondent is asked if anyone in her household has consumed foods from a particular food group in the preceding 7 days, and the number of days on which that food was consumed, followed by prompting about different meals and snacks and examples of locally-available foods, was used. The data were entered into a Microsoft Excel spreadsheet and the food consumption score was calculated following the prescribed method [96, 97]. Descriptive statistics for household food consumption score (poor 0 ≤ 28; borderline 28.5 ≤ 42; acceptable > 42), proportion of households consuming nutrient-dense foods, and frequency of household consumption of nutrient-dense foods were calculated. The threshold for an acceptable household diet was adjusted upwards as recommended by [96, 97]), given frequent consumption of sugar and oil. Frequency of consumption data are disaggregated as 0, 1–3, 4–6 and 7 days (rather than the recommended 0, 1–6 and 7 days) because a high proportion of foods, especially vitamin A rich foods and fruits and vegetables, were reported to be consumed on only 1–3 days of the recall period. While ‘fruits and vegetables’ are not a specific category in the methodology, we present this data to gain a better understanding of consumption of nutrient-dense foods.

#### Household food insecurity access scale

Data on household experiences of food insecurity within the access domain of food security was collected using the module developed by Coates et al. [24]. The module comprises nine condition questions across three domains of food insecurity ((i) anxiety and uncertainty; (ii) insufficient quality; (iii) insufficient food intake and its physical consequences) and asks the respondent about the household’s experience over the last 4 weeks. For each affirmative response, the respondent is asked about the frequency of occurrence of that experience, captured as rarely (1–2 times in the past 4 weeks), sometimes (3–10 times), often (> 10 times). The data were entered into a Microsoft Excel spreadsheet and descriptive statistics for the proportion of households experiencing each condition and domain, HFIAS score (range 0–27), and HFIAS prevalence (food secure, mildly, moderately and severely food insecure) were calculated following the prescribed method [24]. A food secure household experiences none of the food insecurity (access) conditions, or may just experience worry, but rarely; where as a severely food insecure household experiences a reduced quantity of food often, and/or experiences any of the most severe conditions, such as running out of food.

#### Household wealth

Household wealth was evaluated using a Material Styles of Life scale based on the building materials of the house, access to electricity (none, purchased, own source), access to improved sanitation, and the presence or absence of household assets (fan, television, mobile phone, tablet, fridge, couch/sofa set, washing machine, scooter) [42, 80]. These items were factor analyzed using the principal component method, and items with low factor loadings removed [21]. The first principal component axis explained 27.72% of the variation in wealth among households. Households with higher wealth had homes with brick walls and cement floors, their own source of electricity and access to improved sanitation. The scale was weighted by the number of household members and households were grouped into three wealth levels: low (lowest 40%), middle, and high (highest 20%).

Additional analyses were undertaken in SPSS (version 24, IBM). Seasonal differences between mean household food consumption and HFIAS score were explored with paired t-tests. Seasonal differences between the proportion of households in each food consumption score and HFIAS category were explored using Wilcoxon signed-rank tests. Seasonal differences between the frequency of consumption of key food groups and of foods within these food groups were explored using Wilcoxon signed-rank and sign tests. Differences between household’s experience of the HFIAS domains and conditions were explored using McNemar’s tests. Logistic generalized linear mixed models (binomial with logit link, with household as a random intercept and variance component as covariance structure) were tested to assess the association between severely food insecure households and different socio-demographic factors, with a multivariable model developed with confounders and variables with *p* < 0.1 from the univariable model included as predictors. Model diagnostics for the final multivariable model included a low Akaike Information Criterion and residual diagnostics, model convergence and absence of multicollinearity as indicated by variance inflation factors (VIF = 1.0). Two-tailed *p* values < 0.05 were considered significant.

Transcripts of the interviews and focus group discussions, along with field notes, were entered into NVivo Pro 12 (QSR). Review and analysis of data occurred parallel to the extended period of field work, allowing for refinement and extension of research activities to explore emerging themes [81]. We applied content analysis to systematically analyze textual data and interpret its meaning [31]. The first author reviewed each data source numerous times to ensure familiarity and then coded the data deductively using an unconstrained categorization matrix based on themes (categories) and sub-themes drawn from the research framework [32] (see Supplementary Information: Table S1). Emergent sub-themes were added to the matrix as necessary. A structured approach was applied in which data were coded by sets of themes (i.e. multiple pass), data within categories was compared, then linkages between themes and sub-themes were evaluated [81]. This approach was appropriate given that the broader aim of the research project was to assess evidence of the contribution of fish and small-scale fishing livelihoods to food and nutrition security along Kawarazuka and Bene's [58] three pathways [32]. Anonymized representative participant quotes (identified by reference code) are provided to evidence perceptions of and responses to food insecurity [31].

## Results

### Characteristics of households

The average age of female respondents to the household survey was 29.7 years (SD +/− 6.7 years) (Table 2). Nearly half of the women (48%) had not completed primary school, and 22.7% had attempted or completed a higher level of education. Households, comprising a collection of people who regularly eat together and sleep under the one roof [50], typically comprised between 4 and 6 members (59% of all households) (excluding children who were boarding away from home). Larger households generally comprised a younger couple with children living with one set of parents, in which case the older female retained responsibility for management of household monies and food provision and preparation, with the younger couple contributing towards household expenses and labor.

**Table 2:** Socio-demographic characteristics of female respondents and households

Characteristics	Mean ± SD or n (%)	N
*Maternal characteristics*		
Maternal age, years	29.7 ± 6.7	66
Highest level of schooling completed		66
Some primary	32 (48.5)	
Completed primary (6 years)	19 (28.8)	
Some or completed secondary, or further education	15 (22.7)	
Mother has income-generating livelihood activity	26 (39.4)	
*Household characteristics*		
Number of household members	4.69 ± 1.75	66
Household livelihood activities (no.)		66
All livelihood activities	2.24 ± 1.08	
Small-scale fisheries livelihood activities	1.55 ± 0.9	
Women’s livelihood activities	0.45 ± 0.41	
Other non-fishing livelihood activities	0.24 ± 0.56	
Material Styles of Life scale (wealth)	0, ± 1 Range − 1.21805, 2.24643	66
Lowest wealth	24 (36.4)	
Middle wealth	30 (45.5)	
Highest wealth	12 (8.2)	
Double burden households ^a^	9 (16.98)	53
Recipient of RASTRA ^b^	14 (21.2)	66

^a^ households were classed as ‘double burden’ when the mother has a BMI class of overweight or obese and the child has stunted growth; anthropometry data was not collected from 3 women who disclosed a pregnancy and 10 children; ^b^ RASTRA is an Indonesian Government program providing supplemental rice for poor households

The average number of income-generating livelihood activities per household was 2.24 (SD ± 1.08; range 1–5), with 43% of households pursuing two or more income-generating livelihood activities. Men’s livelihood strategies were centered on capture fishing and fish trading activities, with only 4.5% of households not engaged in small-scale fisheries activities. Men participated in ten different capture fisheries livelihood activities, with fishing practices varying according to the target species and gear used. Women in 41% of households reported pursuing an income-generating livelihood activity, typically home-based activities such as making and selling traditional cakes. Other women took up community roles such as volunteer health workers and received a small monthly incentive payment.

Approximately 17% of households were double burden households, with the mother classed as overweight or obese and an index child having stunted growth.

### Consumption of nutrient-dense foods

**Fig. 1:**
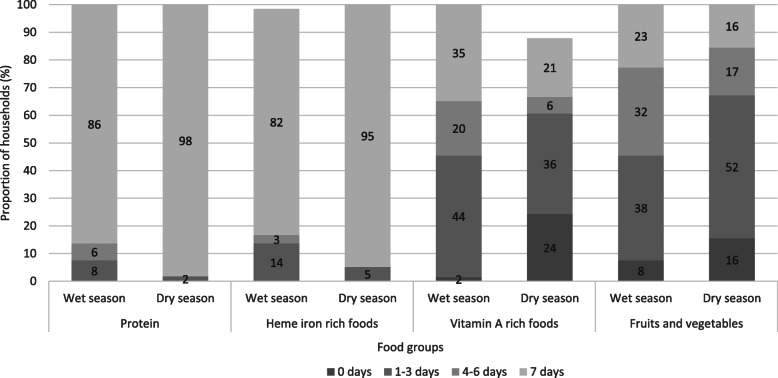
Proportion of households consuming nutrient-rich food groups in 7-day recall periods, by frequency category. Figure 1 shows that most households were frequent consumers of protein and iron-rich food groups, and less frequent consumers of vitamin A rich foods, and fruits and vegetables

**Fig. 2:**
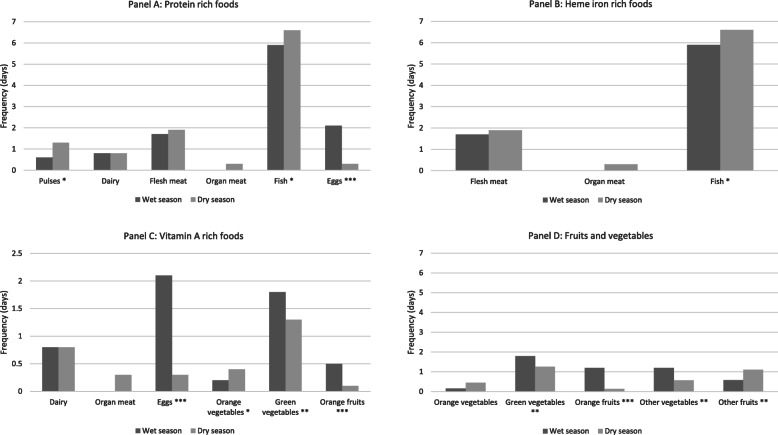
Average reported frequency of consumption of nutrient-rich food groups: protein, heme iron, vitamin A, and fruits and vegetables. The panels in Fig. 2 show the average reported consumption frequency of foods within nutrient-dense food groups by households during the seven-day recall periods. Fish is the most frequently consumed protein and heme iron rich food, while eggs are an important source of vitamin A. Dark green leafy vegetables are an important source of vitamin A and the most frequently consumed food group among fruits and vegetables. Notes: *n* = 66 wet season; *n* = 59 dry season; * *p* < 0.05, ** *p* < 0.01, *** *p* < 0.001; there were statistically significant median seasonal differences in the reported consumption of pulses (z = 2.58, *p* = 0.010), fish (z = 2.22, *p* = 0.027), eggs (z = − 6.17, *p* < 0.0005), orange vegetables (z = 2.09, *p* = 0.027), dark green leafy vegetables (z = − 2.59, *p* = 0.010), other vegetables (z = − 2.86, *p* = 0.004), orange fruits (z = − 4.97, *p* < 0.0005) and other fruits (z = 2.83, *p* = 0.005)

Most households appear to have acceptable access to adequate food, with 89 and 97% of households having an ‘acceptable’ food consumption score for the wet and dry season respectively (wet season: *M* = 54.95, *SD* ± 13.88, range: 22.5–85; dry season: *M* = 58.89, *SD* ± 10.98, range: 29–84). The mean food consumption score was higher in the dry season (3.681, 95% CI [0.022, 7.340]). However, when the consumption frequency of nutrient-dense food groups is disaggregated, we found that most households consumed protein and iron rich foods every day, and vitamin A rich foods less frequently (Fig. 1). Protein-rich foods, principally fish, were consumed on all 7 days of the seven-day recall period by 86% of households in the wet season and 98% of households in the dry season (Fig. 2a). Consumption of heme iron rich foods was also frequent, with 82% of households consuming heme iron rich foods on all 7 days of the seven-day recall period in the wet season and 95% of households in the dry season. Again, fish was the most important source of heme iron in household diets (Fig. 2b). Consumption of vitamin A rich foods was less frequent, with only 35 and 21% of households consuming vitamin A rich foods on all 7 days of the wet and dry season seven-day recall periods. Conversely, a higher proportion of households consumed vitamin A rich foods on only 1–3 days of the respective wet and dry season seven-day recall periods. The most commonly consumed vitamin A rich foods were eggs and dark green leafy vegetables (Fig. 2c). Consumption of vitamin A rich foods was likely higher in the wet season because eggs were used as a substitute when fish were not available and were provided to young children when only a non-preferred species of fish (e.g. squid) was available. Overall consumption of fruits and vegetables was very low in both the wet and dry seasons (Fig. 2d). Collectively, consumption of vitamin A rich foods (*z* = − 4.26, *p* < 0.0005) and fruits and vegetables (*z* = − 2.27, *p* = 0.023) were statistically significantly higher in the wet season.

### Perceptions of food insecurity experience

**Fig. 3:**
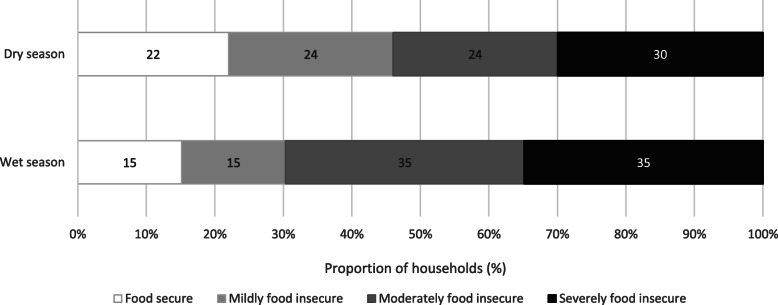
Proportion of households experiencing food insecurity during the wet and dry seasons. Figure 3 shows the proportion of households experiencing the different degrees of food security, as determined by reported severity of experience of the different conditions

Many of the households surveyed experienced varying degrees of food insecurity. Only 15 and 22% of households were classed as food secure during the wet and dry seasons respectively (Fig. 3). Around one third of households were classed as severely food insecure in both seasons, with an additional third of households moderately food insecure in both seasons. Overall, reported experiences of food insecurity were higher in the wet season (*z* = 2.24, *p* = 0.025).

**Fig. 4:**
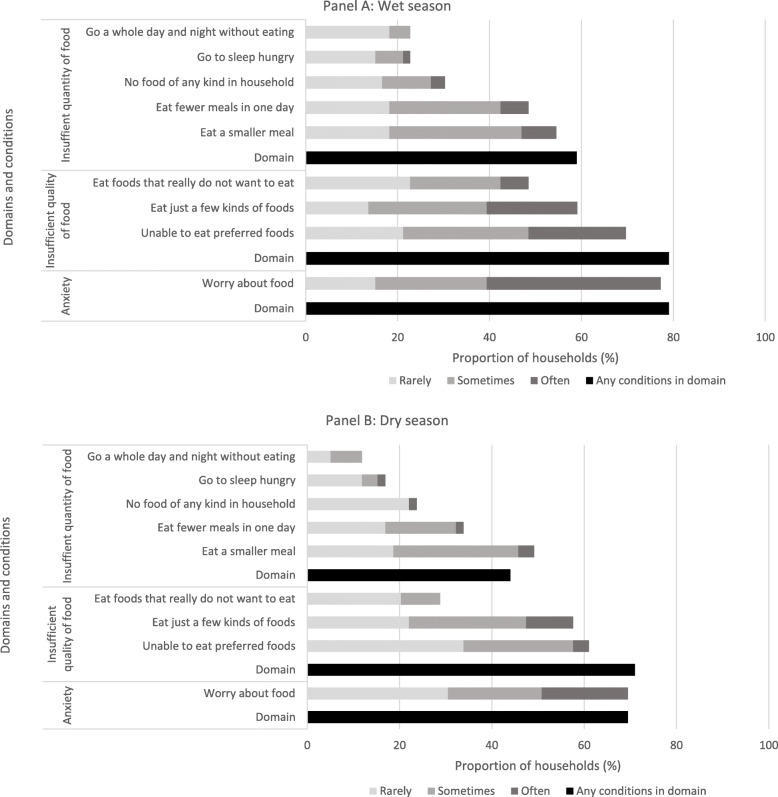
Proportion of households experiencing different domains and conditions of food insecurity in the wet and dry seasons. Figure 4 shows the proportion of households experiencing the domains of food insecurity (access) (all) and the proportion of those households experiencing the condition of food insecurity (access) within each domain. While many households reported experiencing different domains of food insecurity in both the wet and dry season, the severity of experience was higher in the wet season, with households more likely to experience anxiety and worry about food, and insufficient quality of food

A majority of households reported experiencing the domains ‘anxiety and worry’ about access to food (79% wet season, 69% dry season), and an ‘insufficient quality of food’ (79% wet season, 71% dry season) (Fig. 4). In interviews numerous women said they worried about securing food; for example, *we often worry* (IF9-FS1-2017); *worrying about your children, whether they have something to eat or not* (IF6-FS1-2017). The proportion of households that reported experiencing the different conditions of food insecurity was higher in the wet season. Overall, household perception of severity of experience of food insecurity was lower in the dry season, with a smaller proportion of households reporting experiencing the different conditions *often* (i.e. more than 10 times over the last 30 days). Reported frequency of experience of ‘worry or anxiety’ (*z* = − 3.39, *p* = 0.0001), having to ‘eat foods that really did not want to eat’ (*z* = − 3.09, *p* = 0.002), ‘eat fewer meals in one day’ (*z* = − 2.19, *p* = 0.029), and having ‘no foods of any kind in the household due to lack of resources’ (*z* = − 1.97, *p* = 0.048) were all significantly higher in the wet season.

### Factors affecting household perceptions of food insecurity experience

The association between a household being severely food insecure and numerous independent variables was assessed: maternal characteristics (age, level of education, livelihood, mother having adequate dietary diversity), household characteristics (number household members, number livelihood activities, material style of life score, double burden household, household receives RASTRA), and season. The final model showed that the strongest predictors of households being severely food insecure were mother having low education (not having completed primary school) and households having a low MSL score (i.e. having low material wealth) (Table 3).

**Table 3:** Association between a household being severely food insecure and explanatory factors

Factor	Univariable model			Multivariable model		
	Odds ratio	95% CI	*p* value	Odds ratio	95% CI	*p* value
Maternal age (years)	0.9	0.9–1.0				
M hasn’t completed primary school	0.3	0.1–0.6	0.005 **	3.8	1.5–9.9	0.007 **
Mother has livelihood activity	1.1	0.4–2.9				
Mother has adequate dietary diversity	1.0	0.4–2.8				
No. household members	1.1	0.8–1.5				
No. household livelihoods	1.3	0.8–2.1				
Material Styles of Life score (scale)	0.5	0.3–0.8	0.011 *	0.5	0.3–0.9	0.020 *
Double burden household	0.5	0.1–2.7				
Household receives RASTRA	3.3	1.1–10.0	0.039 *			
Season	0.8	0.4–1.5				

*CI* Confidence Interval; *p* value only shown when significant; * p < 0.05 and ** p < 0.01

### Responses to food insufficiency

Households employed a range of immediate and short-term consumption strategies and longer-term non-consumption strategies in anticipation of or in response to a shortfall in food (Table 4). The primary consumption strategies employed were changes in diet and increasing short-term food supply. Women responded to a shortfall in food by purchasing cheaper and less-preferred foods. As one woman from field site 1 explained: *if there is no money, we eat noodles and eggs, because the fish here are expensive, the vegetables are also expensive* (IF9-FS1-2018). In field site 2 some women *buy dried cassava from a seller from Ende and alternate with rice in meals* (FG1-FS2-2017); another woman explained: *if [I] don’t have rice, I grind down corn to make porridge or boil and mash banana* (HSF4-FS2-2017). Women also used their social capital to secure food from friends, neighbors and relatives; sharing of excess fish was common, and one woman revealed: *yesterday my sister brought a large bowl of vegetable soup that had pumpkin and carrot in it and my family shared it* (HSF1-FS2-2018). The purchasing of food on credit was also commonplace. Women obtained staple and other foods (e.g. flour, sugar) on credit from their husband’s fishing boss, whom they described as stockpiling staples during the wet season, and other kiosk vendors. One third of households who had obtained loans from a boss had done so to secure food or meet other general household expenses. One woman explained: *my ability is that if we have not enough money, I will try to owe the stall [and] I do selling cakes to cover our lack of money … I will owe to someone; I don’t care what people say, they don’t know the situation* (IF6-FS1-2017). The ability to obtain fruits and vegetables on credit from village vendors was identified by women as one of the reasons for not obtaining fruit and vegetables from the larger markets in Labuan Bajo. Resort to wild foods, such as gleaning for marine resources, was not common despite the maritime traditions of the Sama-Bajau. Women did occasionally harvest ‘*kelor*’ (*Moringa oleifera*) when they could not obtain other leafy greens: *moringa leaves grow here so that would be our only vegetable* [in the wet season] (IF4-FS1-2017). Overall, in times of food insufficiency women produced simpler less-nutritious meals comprising mainly rice or a substitute staple, with a small (or no) accompaniment. As one woman explained: *sometimes [we] will just eat rice for a meal, when don’t have fish or vegetables* (HSF1-FS1-2017).

**Table 4:** Strategies employed by households in response to food insufficiency

Type of strategy	Examples
*Consumption strategies*	
Change diet	Consume cheaper / less-preferred foods, such as noodles and egg instead of preferred rice and fish
Increase short-term food supply	Borrow (or in kind) food from friends, neighbors or relatives
	Purchase food on credit from boss or kiosk owner
	Glean for marine resources
	Harvest locally-growing *kelor* (*Moringa oleifera*)
Reduce number of people to be fed	Send children to relatives’ homes for main meals
Ration food supplies	Reduce portion size for all family members
	Reduce maternal portion size (eat only after other family members)
	Skip one or more meals in a day
*Non-consumption strategies*	
Alteration of income-earning patterns	Fisher abandons income-earning fishing activity to ensure he harvests fish for household consumption
	Female household member borrows money to establish a home-based food or trading business
Sale of small assets	Sell small livestock (i.e. goats)
	Sell or pawn gold jewelry
Savings	Use cash savings accrued during dry season

Households employed several non-consumption strategies. Fisher-husbands altered or re-directed their fishing practice while at sea depending on their success in harvesting higher-value export fish. If fishers had been unsuccessful, and hence had generated no income, they would re-direct their fishing effort to the harvest of small reef fish so as to ensure they were able to provide fish for their family. A fisher explained: *the catch is uncertain, if the blessing is willing then I would have a lot of catch, but if the blessing is not willing, even for consumption would be hard to find* (IM3-FS2-2018). Women also sought to increase their ability to access food by establishing home-based food or trading businesses. These small informal businesses were typically established with food stuffs or goods obtained on credit, and thus the women risked falling into debt. For example, one woman explained: *I also sell some things which could be paid by installment... I see that as a business opportunity and then settle up the installment details … the payment could be settled up to two or 3 years after my merchandise is sold* … *I see a lot of people running the exact business as I do and I think I am the most patient creditor* (IF2-FS1-2017). Around two-fifths (43.6%) of households kept free-roaming chickens or goats which could be sold for cash when required. Less than one third (29%) of households accrued savings (in the form of cash or gold jewelry) from their fishing and other business activities which could then be relied upon in times of food insufficiency.

## Discussion

This study presents findings from an exploratory assessment of food and nutrition security in three specialised fishing communities in Komodo District, NTT, eastern Indonesia. We found that households appear at first to have adequate access to consumption of diverse food. However, the typical household dietary pattern comprised frequent consumption of the staple rice and regular consumption of fish, and only occasional consumption of vitamin A rich foods and fruits and vegetables in general. Fish were the primary dietary source of protein and heme iron; a wide range of fish were consumed, including reef-associated finfish such as emperor, rabbitfish and grouper, and small pelagics such as anchovy and sardines. Studies indicate that fish can be an important dietary source of vitamin A [11, 85]. However fish are not included in the ‘vitamin A rich food group’ used to construct the Food Consumption Score for nutritional quality analysis, perhaps reflecting the indicator’s historical development in Sub-Saharan Africa where per capita fish consumption is among the lowest in the world [38]. The inclusion of fish is hampered by complexity arising due to the form, content and bioavailability of vitamin A differing among fish species and also affected by a range of ecological processes [16, 84]. Despite recent efforts in other countries [11, 79, 83], there is limited nutritional data available for commonly consumed fish in Indonesia. The *Food Composition Table for Indonesian* lacks sufficient detail, listing only a handful of fish (for example ‘anchovy’, ‘fish’, ‘fish, sea’ and ‘fish, tuna’) and fish dishes, although over 870 species of bony fish are marketed [89, 99]. This lack of information, applying equally to other key micronutrients and minerals such as iron and zinc, is compounded by limited understanding of the impact of post-harvest handling and processing and cooking methods on the nutrition quality of fish. In the case study communities, fish were observed being processed using methods that raised food safety concerns, further highlighting the complex interplay of factors associated with ensuring access to adequate, nutritious and safe foods for all.

Household consumption of fruits and vegetables in the communities studied varied seasonally in accordance with local availability and corresponding affordability. A small number of nutrient-dense moringa trees (*Moringa oleifera*, *kelor*) were grown by individual households in each community. Moringa appear to be underutilized as a versatile and nutrient-dense food source, particularly given that little else was successfully cultivated locally. Fruits were considered as a snack food whereas vegetables were typically consumed in small portions as a side dish or incorporated sparingly in stir-fried dishes or soups. This appears consistent with other dietary patterns across Indonesia, with data at the national level indicating that Indonesians consume less than half the recommended daily amount of fruits and vegetables [9, 94]. Infrequent consumption of fruits and vegetables were also evident in the 24-h recall dietary quality data we collected for women of reproductive age and infants and young children [45]. Analysis of this data found that both the mother and child in one half of mother-child pairs surveyed had consumed fruits and vegetables in the 24-h recall periods (53% in the wet season and 49% in the dry season) and, that in 88% of mother-child pairs neither the mother nor child had consumed vitamin A rich fruits or vegetables. Other studies from Indonesia have shown that diets lacking diversity increase the risk of vitamin A and iron deficiency for infants and young children, female adolescents, and women of reproductive age, suggesting that women and children may be susceptible to micronutrient deficiencies despite the frequent consumption of fish [8, 64, 86]. Moreover, as is also observed in Albert et al. [1]‘s study in the Solomon Islands, the dietary quality data contrast with the initial impression given by the Food Consumption Score. This suggests that the cutoff scores used for poor, borderline and acceptable diets in contexts where there is frequent consumption of fish (and other protein foods) should be adjusted further upwards. This anomaly reinforces the importance of selecting an appropriate combination of food and nutrition security indicators, depending on the purpose of the study and feasibility of data collection given available resources and other constraints [37, 61].

A healthy diet encompasses a balanced, diverse and appropriate selection of foods, ensuring that the need for macro- and micronutrients are met given a person’s gender, age, physical activity level and physiological state [40]. Increasing dietary diversity is one of the core messages of Indonesia’s revised dietary guidelines [75] and is included as an objective of the food-related policy framework (*National Medium-Term Development Plan 2015–2019*, *Strategic Policy for Food and Nutrition 2015–*2019, *National Action Plan for Food and Nutrition 2017–20*19). While recent progress has been made with respect to increasing the consumption of fish [2], consumption of fruits and vegetables declined by around 4 % between 2012 and 2016 and continues to fall, in line with dietary transitions occurring in many low- and middle-income countries [94]. There is a substantial difference in the frequency of consumption of fruits and vegetables between Indonesian households in the lowest and highest income quintiles [94]. A recent assessment found that 38% of Indonesian households could not afford a staple-adjusted nutritious diet, increasing to 68% of households in NTT Province where nearly 60% of rural households’ monthly expenditure is on food [12, 98]. Low levels of fruit and vegetable consumption have been reported in small island developing states across the Indo-Pacific region, largely due to cost and affordability but also changing taste preferences, convenience and environmental challenges [1, 19, 33, 57]. Availability and affordability are key factors influencing household purchasing decisions, with low income households tending to focus on consumption of sufficient energy-dense staple foods to meet calorie needs [88]. Efforts to increase dietary diversity are also influenced by taste preferences, food habits and taboos, low nutritional awareness, convenience, food safety concerns and changing lifestyles [56, 88]. We also found that women served other members of their household before themselves and relied upon the advice of women elders and traditional midwives rather that community health personnel [45]. These factors can lead to inequalities in intra-household distribution of nutrient-dense foods, which can be masked by studies at the household level [49]. There is thus a critical need to further explore and address the barriers to consumption of nutrient-dense foods in the communities in this study area.

The households included in our study reported high levels of perceived food insecurity, with household perceptions of food security significantly higher in the dry season. Food insecurity was likely higher in the wet season due to poor weather conditions which kept fishers ashore resulting in there being less fish harvested for subsistence consumption or sold for income. Poor weather also meant that small fruit and vegetable vendors could not travel between the islands and mainland markets. Only a small proportion of households reported the more severe food insecurity condition experiences, such as ‘not having food of any kind in the household’ and ‘going a whole day and night without eating’, although these were rare occurrences. Fabinyi et al. [35] identified similar experiences in fishing communities in the Philippines. A higher level of food insecurity was associated with a low level of maternal education and lower material styles of life score, a proxy for household wealth. These factors have previously been identified as risk factors for food insecurity and children’s micronutrient deficiencies [48, 90]. Empowering women through education is argued to be the single most important determinant of food security, with women with higher levels of education likely to delay having children, have increased opportunity to participate in the work force, greater decision-making capability and the ability to influence household purchasing to support improved nutrition outcomes [55, 90]. Darling [27] and Fiorella et al. [43] found household wealth was associated with higher levels of food security in inland and coastal fishing communities in Kenya respectively, irrespective of the household pursuing fishing livelihoods.

In these specialised fishing communities, food insecurity was a cyclical and seasonal phenomenon, with the flow of fish, income and food linked to the type of fishing activity pursued. For many households, food insecurity reflected the lunar cycle, with night fishing practices such as spearfishing and lift-net fishing significantly reduced or ceasing during each full moon phase. Households employed a range of consumption and non-consumption strategies to manage through times of food insecurity; these include four commonly identified coping strategies: dietary change, food-seeking (including borrowing, purchasing on credit and reliance on wild foods), household structure, and rationing [65, 66]. However the strategies were not “short-term responses to an immediate and inhabitual decline in access to food” [28], but rather routine insurance and adaptive strategies in response to the vagaries of the small-scale fisheries activities pursued and the broader socio-economic context. The income generated by fishing, particularly for poorer fishing households without fishing capital (boats, engines, gear), was too low to allow for savings, leaving the fisher and his household reliant on patron boat owners for work and other support such as loans to cover food or medical expenses [35, 41, 73]. While numerous studies have noted the importance of livelihood diversification strategies in increasing household resilience [47, 71], such options are limited in specialised fishing communities by the pervasiveness of patron-client relationships, geography, community size and cultural identity associated with fishing as a ‘way of life’ [67]. None of the households in the study communities engaged in cropping, kitchen gardens were uncommon, soils were poor and fresh water limited. Some fisher-wives created small food-based or petty-trade businesses, however, the vagaries of fishing and the flow of money within the communities impacted these businesses too, leaving the women vulnerable to debt. The range of strategies pursued by households highlights their high level of risk to entitlement failure, and vulnerability to continued food insecurity.

### Strengths and limitations

This research places a spotlight on the experiences of food and nutrition security in remote rural specialised fishing communities, a vulnerable group which has been overshadowed by a consistent focus on food security in agricultural small-holder households. Several limitations should be considered. As a small cross-sectional case study, the results relate to households in these communities comprising a woman of reproductive age and a child aged between 6 months and 5 years, and therefore may not be generalizable to the wider Indonesian population. The analysis drew on maternal-reported food consumption and experience of food insecurity data which may be subject to recall error and, despite careful explanation of the purpose of the study, social desirability bias. Consumption of fruits and vegetables are driven by seasonal availability and greater access due to lower prices. While we collected data in periods covering the major seasons from a fisheries perspective, neither survey period coincided with the local mango season which occurs from October to December. A repeat survey during that period may have produced elevated food consumption and dietary diversity scores due to more frequent consumption of mango, a vitamin A rich fruit. We acknowledge that the quality of food consumption data collected using questionnaires is imperfect and that quantitative dietary assessment with additional biochemical measures would allow for a more accurate assessment of nutrition status. Finally, research activities were undertaken by a multi-lingual field research team, so our methodology incorporated frequent cross-checking and the use of probing questions to clarify understanding and further explore responses. Transcripts were transcribed verbatim and checked for correspondence with contemporaneous notes, but unfortunately resources and timing did not allow for rechecking of transcribed material with research participants. While data were not co-coded, regular and reflexive debriefing supported bracketing.

## Conclusion

This study assessed food and nutrition security in three specialised fishing communities in Komodo District, eastern Indonesia. We found that while households appeared to have access to diverse foods, and fish were important dietary sources of protein and heme iron, consumption of nutrient-dense fruits and vegetables was infrequent, leaving households vulnerable to micronutrient deficiency. We also found that households reported experiencing high levels of food insecurity, escalating in the wet season. Household members responded to shortfalls in food by employing a range of insurance and adaptive strategies, however these were nutritionally and economically unsustainable [28]. These findings highlight the vulnerability of specialised fishing communities, particularly given their linkages to international markets and the potential impacts of a changing climate. Enhancing food and nutrition security in specialised fishing communities requires a culturally-sensitive cross-sectoral response that strengthens livelihood opportunities, supports and/or provides improved financial literacy and affordable credit, and supports local production of higher-value fish-based foods and increased access to nutrient-dense fruits and vegetables.

## Supplementary Information

**Additional file 1:** **Table S1.** Outline of research framework, with themes and sub-themes used to code and analyse data.

## Data Availability

The datasets used and/or analysed, and the interview and discussion guides used, during the current study are available from the corresponding author on reasonable request.

## Abbreviations

HFIAS: Household Food Insecurity Access Scale
NTT: Nusa Tenggara Timur
RT: *Rukun tetangga* (Bahasa Indonesia), neighborhood group

## Footnotes

1 From the June to September, in the dry season (*musim timur*), the prevailing wind blows from the east, bringing little moisture and calmer seas conditions. From December to March, in the wet season (*musim barat*), the prevailing wind blows from the west, bringing higher rainfall and stormier weather. Transitional conditions occur in the remaining months. In 2018, the dry season was well established by early March.
