# Supplementary material for: Coping or adapting? Experiences of food and nutrition insecurity in specialised fishing households in Komodo District, eastern Indonesia
Source: BMC Public Health. 2021 Feb 15;21:355. doi: 10.1186/s12889-021-10248-3 (PMC7885255; doi:10.1186/s12889-021-10248-3)
Supplement: Supplementary file 1 — Additional file 1: Table S1. Outline of research framework, with themes and sub-themes used to code and analyse data. [file 12889_2021_10248_MOESM1_ESM.docx]

Supplementary Information

Table S1: Outline of research framework, with themes and sub-themes used to code and analyse data

| ***Causes of nutritional status*** | ***Theme*** | | ***Sub-theme*** | ***Linking or additional sub-themes*** |
| --- | --- | --- | --- | --- |
| Immediate causes | Dietary intake | | - MDDW |  |
|  |  |  | - CDD |  |
|  |  |  | - FCS-N | - Access to nutrient-dense foods |
|  |  |  | - No. IYC meals per day | - Food taboos for children and women |
|  | Disease | | - IYC illness in past 2 weeks |  |
|  |  |  | - Treatment for intestinal worms |  |
|  |  |  | - Disease prevalence |  |
| Underlying causes | Household food security | | - HFIAS | - Coping / adaptation strategies   … Anxiety  … Borrowing  … Link to patron-client relationship |
|  |  |  | - Seasonal change in HH FNS | - Characteristics of wet and dry season   … link to livelihood activities |
|  |  |  | - Household wealth | … link to assets |
|  |  |  |  | - Recipient of RASTRA / BPNT |
|  |  |  | - Sources of food |  |
|  |  |  | - Ownership of home garden and livestock |  |
|  | Improving care for children and women’s health | Care for women | - Roles of women in household and community | - Aspiration for self and children |
|  |  |  |  | - Empowerment (achievements, agency, enabling resources) |
|  |  | Breastfeeding and IYC feeding practices | - Breastfeeding duration, introduction of complementary foods | - Sources of information |
|  |  |  | - Intra-household distribution of food (women, IYC) | - Fish - Other nutrient-dense foods |
|  |  | Psychological-social stimulation of children and support for their development | - Roles of children in household |  |
|  |  | Food preparation and food storage practices | - Food system | - Household meal pattern |
|  |  | Hygiene practices | - Knowledge of hygiene practices |  |
|  |  | Care of children during illness | - Illness in past two weeks |  |
|  | Health service and health environment | Health services | - Access to health services, esp. maternal and child health |  |
|  |  | Access to clean water | - Sources and treatment of drinking and other water |  |
|  |  | Environmental sanitation | - Toilet facilities |  |
|  |  |  | - Disposal of waste |  |
| Basic causes | Household assets | Financial assets  Human assets  Natural assets  Physical assets  Social assets | - Assets required or used for livelihood strategies | - Livelihood activity – woman, man |
|  |  |  | - Access to and use of financial services | - Patron-client relationship |
|  |  |  | - Changes and challenges in undertaking livelihood activities | - Vulnerability   … barriers  … weather / season |
|  | Economic structure | Infrastructure to urban markets | - Communications, preservation/storage, transport | - Cool storage chain |
|  |  | Access to local markets | - Location and trade in local markets | - Frequency of travel to markets |
|  |  | Post-harvest technologies | - Processing activities and methods | - Source of knowledge, equipment |
|  | Political and ideological superstructure | Sustainable resource management | - Management of coastal zone |  |
|  |  |  | - KNP zonation and permitted activities |  |
|  |  | Policies ensuring access to common-pool resources, credit, health and nutrition, social security, etc | - National development plans | - Government assistance received by community or individuals |
|  |  |  | - Laws, regulations, ministerial decrees |  |
|  |  |  | - International agreements and declarations |  |

*Notes:* *CDD – Children’s Dietary Diversity; FCS-N – Food Consumption Score for Nutritional Analysis; FNS – food and nutrition security; HH – household; HHS – household survey; IYC – infant and young child; KNP – Komodo National Park; MDDW – Minimum Dietary Diversity for Women of Reproductive Age..*
